# UBE3A activates the NOTCH pathway and promotes esophageal cancer progression by degradation of ZNF185

**DOI:** 10.7150/ijbs.61117

**Published:** 2021-07-13

**Authors:** Zhikun Zheng, Bin Zhang, Haixin Yu, Shoukang Li, Naicheng Song, Xin Jin, Jinsong Li

**Affiliations:** 1 Department of Thoracic Surgery, Union Hospital, Tongji Medical College, Huazhong University of Science and Technology; 2 Cancer center, Union Hospital, Tongji Medical College, Huazhong University of Science and Technology, Wuhan, 430022, China; 3 Department of Urology, The Second Xiangya Hospital, Central South University, Changsha, Hunan, 410011, China

**Keywords:** UBE3A, ZNF185, NOTCH pathway, esophageal cancer

## Abstract

**Background:** Esophageal cancer is the sixth-most common fatal malignant tumor worldwide. Little is known regarding the genetic drivers that influence targeted therapy outcomes in patients with esophageal cancer. Exploring the pathogenesis of this lethal tumor could provide clues for developing appropriate therapeutic drugs. Ubiquitin-protein ligase E3A (UBE3A) reportedly promotes or suppresses various types of malignant tumors. However, the cancer-related role of UBE3A in esophageal cancer remains unclear.

**Methods:** The relationship of UBE3A with the clinicopathological features of pancreatic tumors was bioinformatically investigated in the TCGA dataset. The protein levels of UBE3A and ZNF185 were assessed by Western blot and immunohistochemistry. The role of UBE3A and ZNF185 in esophageal cancer growth was assessed by MTS assays, colony formation assays, and experiments in mouse xenograft models. The interaction between UBE3A and ZNF185 was investigated by co-immunoprecipitation. The relationship between UBE3A, ZNF185, and NOTCH signaling pathway was explored by Western blot and quantitative real-time PCR.

**Results:** We found that UBE3A was upregulated in patients with esophageal cancer and enhanced the cellular progression of esophageal cancer. Moreover, we found that UBE3A degraded ZNF185 in esophageal cancer. Additionally, ZNF185 suppressed the progression of esophageal cancer by inactivating the NOTCH pathway.

**Conclusions:** These data demonstrated that aberrant expression of UBE3A led to enhanced progression of esophageal cancer through the ZNF185/NOTCH signaling axis. Therefore, UBE3A might be an ideal therapeutic candidate for esophageal cancer.

## Background

Esophageal cancer is the sixth-most common fatal malignant tumor worldwide 1. Most patients with esophageal cancer are diagnosed with advanced disease because of the absence of early symptoms; the 5-year survival rate is < 25% 2. Esophageal cancer is classified into two types: squamous cancer and adenocarcinoma. The incidence of esophageal squamous cancer is decreasing, whereas the incidence of adenocarcinoma is increasing 3. Surgical resection, local radiation therapy, and adjuvant chemotherapy are ineffective for prolonging the long-term survival of patients with esophageal cancer 4. Little is known regarding the genetic drivers of esophageal cancer. To the best of our knowledge, there are no suitable animal models 5; therefore, targeted therapies for esophageal cancer have remained limited. Understanding the pathogenesis of this lethal tumor could provide clues to aid in the development of appropriate therapeutic drugs.

NOTCH signaling pathway is mediated by a conserved receptors (NOTCH1, 2, 3, 4) and ligands (DLL1, DLL3, DLL4, JAG1, JAG2) interaction system 6, 7. After ligands bind to receptors, these receptors were cleaved and released their intracellular domains that would further interact with RBPJ to activate the NOCTH pathway 8. NOTCH pathway is crucial for determining the cell fate and cell differentiation in normal condition 9. It has been reported that NOTCH regulated squamous differentiation in the esophagus 10. It was not surprising that dysregulation of NOTCH signaling is found in esophageal cancer 11. Notably, activated NOTCH1 increased the number of cancer stem cells to enhance the tumor initiation and epithelial-mesenchymal transition in esophageal cancer 12. Therefore, NOCTH pathway might be a candidate target for esophageal cancer.

E3 ubiquitin ligases regulate the ubiquitination of target proteins, thus contributing to the control of various signaling pathways in normal and cancerous cells 13. Ubiquitin-protein ligase E3A (UBE3A, also known as E6-associated protein [E6AP]) belongs to the HECT (i.e., homologous to the E6AP carboxyl terminus) family of proteins 14. UBE3A degrades p53 in papillomavirus-induced cancers and promotes cancer progression 15. Furthermore, UBE3A has a close association with non-virus related cancer 16. The deregulation of UBE3A reportedly promotes or suppresses various types of malignant tumors 17.However, the role of UBE3A in esophageal cancer remains unclear.

In this study, we systematically explore the cancer related role of UBE3A in esophageal cancer. We found that UBE3A was up-regulated in the esophageal cancer, which contributed to the progression of esophageal cancer. We further showed that UBE3A degraded ZNF185 to modulate the growth of esophageal cancer cells and activate the NOTCH signaling pathway. Thus, UBE3A acted as an oncogenic protein in esophageal cancer.

## Material and methods

### Cell lines and cell culture

The human esophageal cancer cell lines Eca-109 were purchased from the Chinese Academy of Science Cell Bank. TE-1 and CaES-17 cells were purchased from Procell life Science&Technology Co.Ltd. (China). TE-1 and Eca-109 cells were cultured in RPMI-1640 (PM150110, Procell, China) plus 10% FBS (164210-500, Procell, China) and 1% P/S (PB180120, Procell, China) maintained at 37 °C with 5% CO2.

### Plasmids transfection and shRNAs infection

Lipofectamine 2000 (Thermo Fisher Scientific, CN) was applied for plasmids transfections following the manufacture's protocol. Flag-UBE3A was cloned into the CMV-MCS-3xFlag-SV40-neomycin vector by GENECHEM (Shanghai, CN). The lentivirus-based control and gene-specific shRNAs, obtained from Sigma-Aldrich, combined with pVSV-G were employed to produce different lentiviral particles in 293T cells through lipofectamine 2000. 24 h post-transfection, the cell culture medium was replaced with fresh RPMI-1640 medium. After 48 h, the cell culture medium containing the viral particles was harvested and added into the culture medium of esophageal cancer cell lines.

### Western blot analysis, co-immunoprecipitation and antibodies

Ethical approval for the use of human tissues (10 pairs of patient-matched esophageal cancer and adjacent non-cancerous tissues) was obtained by the local ethics committee (Tongji Medical College, China). Written informed consent was acquired from all patients before surgery. The procedure of co-immunoprecipitation and western blot analysis were reported previously 18. Briefly, cells were lysed in RIPA buffer (Beyotime, China) and the supernatant containing proteins were incubated with Pierce Protein G Agarose (Thermo Fisher Scientific, USA) and primary antibody or IgG. Then the precipitates were analyzed by immunoblotting.

The following antibodies were used in this study: UBE3A (Cat No. 10344-1-AP, Proteintech; 1:1000 dilution), GAPDH (Cat No.10494-1-AP, Proteintech; 1:10000 dilution), ZNF185 (Cat No. HPA016438, Sigma; 1:1000 dilution), NOTCH1 (Cat No. 20687-1-AP, Proteintech; 1:1000 dilution), NOTCH3 (Cat No. 55114-1-AP, Proteintech; 1:1000 dilution) and JAG2 (Cat No. 19696-1-AP, Proteintech; 1:1000 dilution).

### Tissue microarray and immunohistochemistry (IHC)

Tissue microarray (Cat No. D880101, Bioaitech, CN) and IHC were performed to assess the levels of UBE3A (Cat No. 10344-1-AP, Proteintech; 1:500 dilution) and ZNF185 (Cat No. HPA016438, Sigma; 1:1000 dilution) in esophageal cancer. The IHC score was evaluated as previously reported 18.

### Proximity ligation assay (PLA)

The Eca-109 cells were fixed by the blocking solution following the manufacture's protocol (Duolink in situ-fluorescence, Sigma). Then, the primary antibodies UBE3A (sc-166689, santa cruz biotechnology; 1:400 dilution) and ZNF185 (Cat No. HPA016438, Sigma; 1:200 dilution) were applied to incubated with the cells for 2 h at 37ºC. Then, cells were washed and incubated with PLA probe for 1 h at 37ºC. The Ligation-Ligase was added to cells at 37ºC. 30 mins later, cells were incubated with Amplification-Polymerase solution for 100 mins. The Duolink In Situ Mounting Medium with DAPI was added to cells to take photos under confocal microscope.

### Xenografts assay

Ethical approval was obtained by the Ethics Committee of Tongji Medical College, Huazhong University of Science and Technology for all animal procedures. BALB/c-nude mice (4-5 weeks old, 18-20 g) were obtained from Vitalriver (Beijing, China). Eca-109 cells were transduced with different lentiviral particles. After puromycin selection for 72 h, cells (1×10^7^ per mouse) were subcutaneously injected into the back of mice. The procedure of xenografts assay was described previously. At the study endpoint, the volume and mass of xenografts were measured.

### Statistical analysis

All data are presented as the means ± standard deviation (SD). Statistical significance was determined by student T test, one-way or two-way ANOVA using GraphPad Prism 5 software. *P*-values <0.05 were considered statistically significant. Image J software was used for the gray analysis of the western blot in this manuscript.

## Results

### UBE3A is upregulated in esophageal cancer

**Figure 1:**
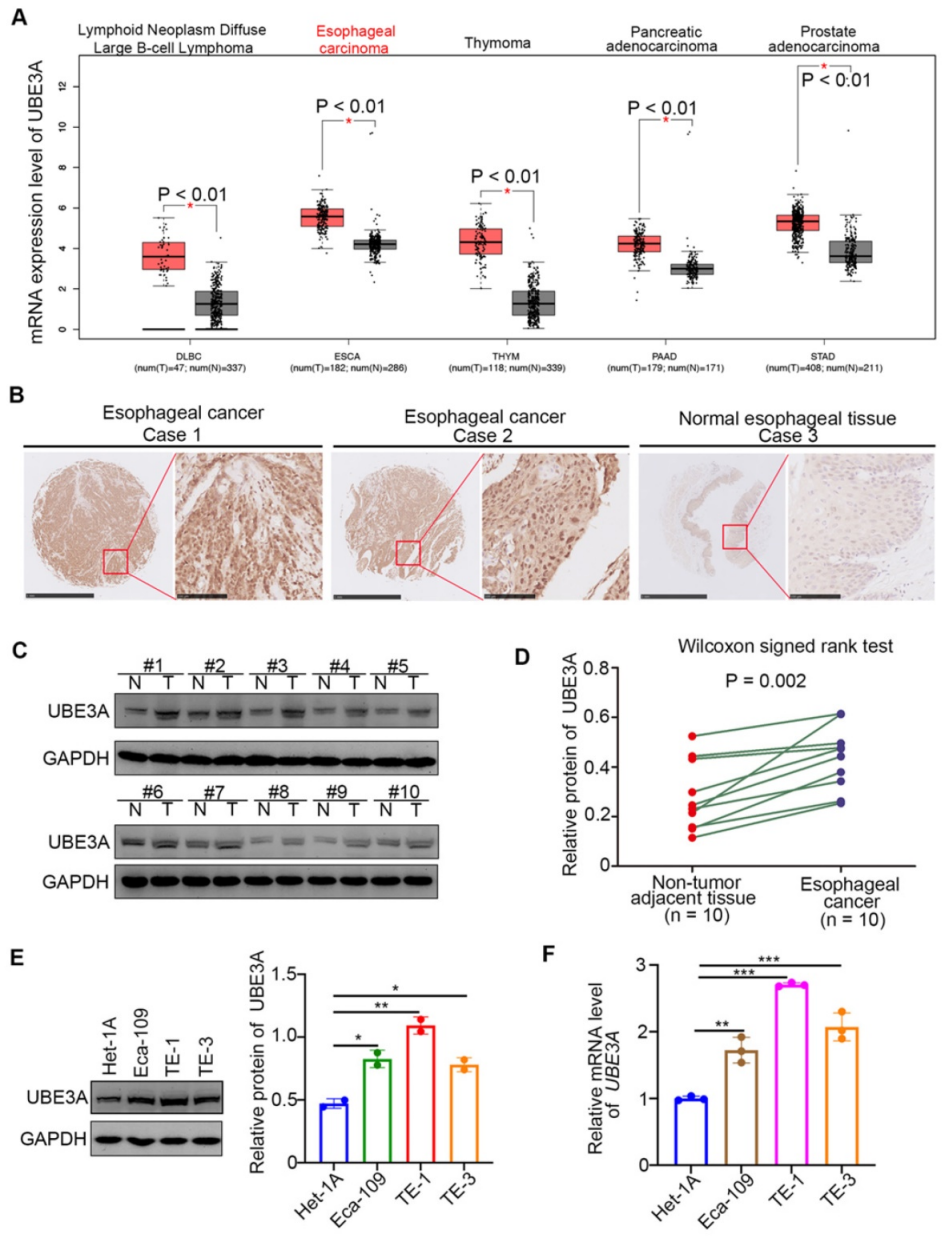
** UBE3A is upregulated in esophageal cancer. A,** analysis the mRNA expression level of UBE3A by using the GEPIA web tool (http://gepia.cancer-pku.cn/). *, P < 0.01. The sample size was indicated in the figure. **B,** representative IHC images stained with UBE3A in the tissue microarray. **C and D,** the protein level of UBE3A from esophageal cancer tissues (n = 10) and adjacent normal esophagus tissues (n = 10) was detected by Western blotting analysis, P = 0.002. The protein levels of UBE3A were quantified by using the Image J software. The quantified value of UBE3A were normalized to the value of GAPDH and indicated in the panel D. **E and F,** Het-1A, Eca-109, TE-1 and TE-3 were harvested for Western blotting analysis (E) and RT-qPCR analysis (F). For panel E, the protein levels of UBE3A were quantified by using the Image J software. The quantified values of UBE3A were normalized to the values of GAPDH and indicated in the panel E. Data presented as Mean ± SD with two replicates. *, P < 0.05; **, P < 0.01. For the panel F, data presented as Mean ± SD with three replicates. **, P < 0.01; ***, P < 0.001.

Analysis of the expression level of UBE3A in cancer tissues, compared with non-tumor tissues, revealed that UBE3A was significantly upregulated in large B-cell lymphoma, esophageal cancer, thymoma, pancreatic cancer, and prostate cancer ( *P* < 0.01) through analyzing the GEPIA web tool (http://gepia.cancer-pku.cn/) (Figure 1A). Notably, the expression level of UBE3A in esophageal cancer was higher than that in large B-cell lymphoma, thymoma, pancreatic cancer, and prostate cancer (Figure 1A). Subsequently, we checked the protein levels of UBE3A in tissue microarrays (TMAs) (Cat No. D880101, Bioaitech, CN) from patients with esophageal cancer (n = 85) by immunohistochemistry (IHC). The UBE3A expression level was elevated in esophageal cancer tissues, compared with normal esophageal tissues (Figure 1B). Similarly, UBE3A protein and mRNA levels were higher in esophageal cancer tissues than in adjacent normal tissues from resected esophageal cancer specimens in our hospital through Western blot analysis and RT-qPCR analysis respectively (Figure 1C, D, Supplementary Figure 1A). Moreover, the mRNA and protein expression levels of UBE3A were elevated in esophageal cancer cell lines (including Eca-109, TE-1 and TE-3), compared with normal esophageal epithelial cells, such as Het-1A. (Figure 1E, F). Overall, these data demonstrated that UBE3A is aberrantly upregulated in esophageal cancer.

### UBE3A contributes to cellular progression of esophageal cancer

**Figure 2:**
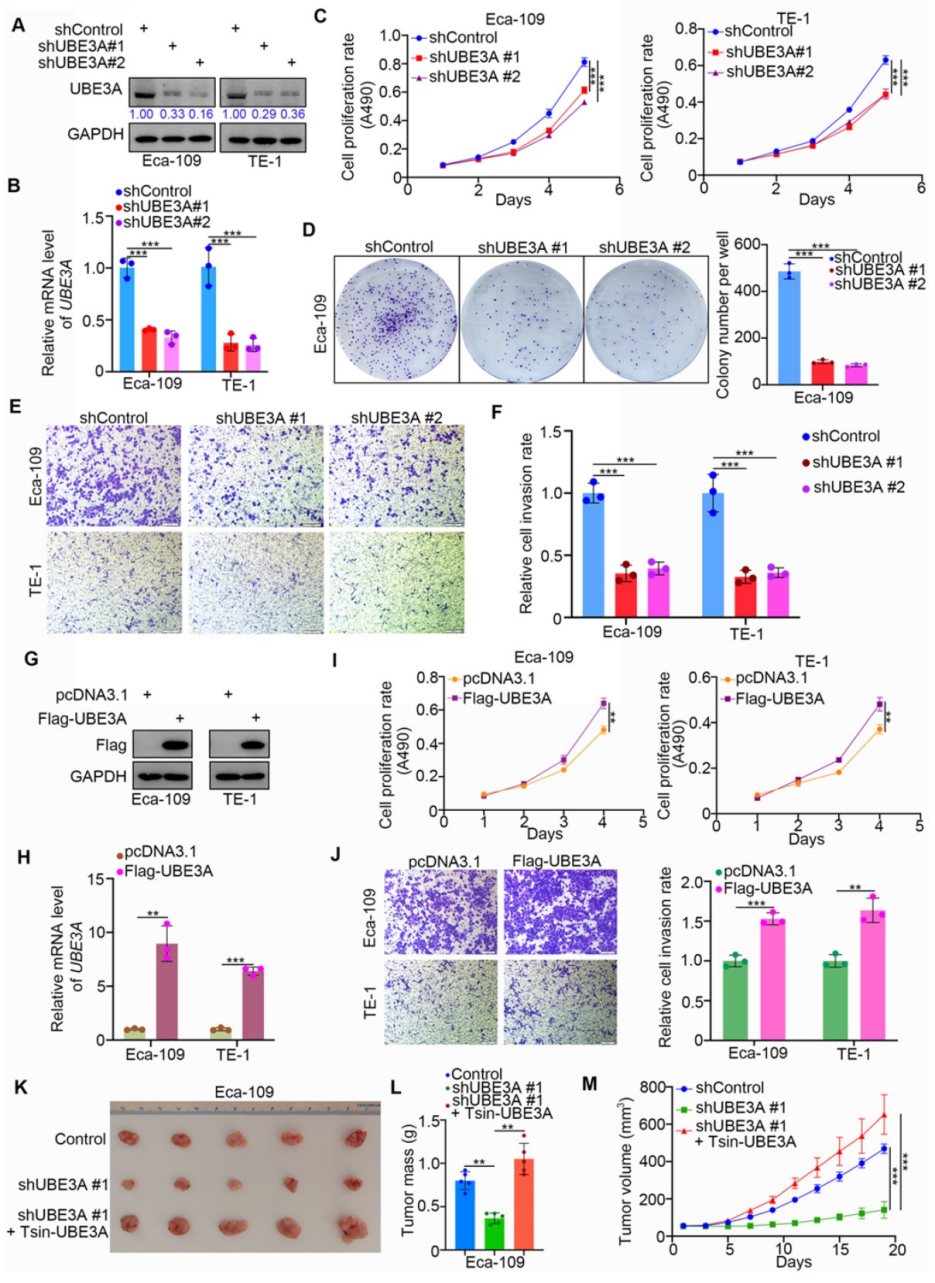
** UBE3A contributes to cellular progression of esophageal cancer. A-F,** Eca-109 and TE-1 cells were infected with indicated shRNAs. 72 h post infection, cells were harvested for Western blotting analysis (A), RT-qPCR analysis (B), MTS assay (C), colony formation assay (D) and transwell assay (E). For the panel B-F, data presented as Mean ± SD with three replicates. ***, P < 0.001. The protein levels of UBE3A were quantified by using the Image J software. The quantified values of UBE3A were normalized to the values of GAPDH and further normalized to the value in cells infected with shControl. **G-J,** Eca-109 and TE-1 cells were transfected indicated constructs. After 24 h, cells were harvested for Western blotting analysis (G), RT-qPCR analysis (H), MTS assay (I) and transwell assay (J). For the panel I-J, data presented as Mean ± SD with three replicates. **, P < 0.01; ***, P < 0.001. **K-M,** Eca-109 cells were infected with indicated shRNAs and Tsin. After 72h puromycine selection, cells were harvested and subcutaneously injected into nude mice for xenografts assay. The image of tumor was shown in panel K. The tumor mass was demonstrated in panel L. The tumor growth curve was indicated in panel M. Data presented as Mean ± SD with five replicates. ***, P < 0.001.

We systematically investigated the role of UBE3A in esophageal cancer. We first achieved stable UBE3A knockdown by lentivirus-based short hairpin RNA (shRNA), using the Eca-109 and TE-1 esophageal cancer cell lines (Figure 2A, B). Notably, UBE3A knockdown markedly reduced the proliferation capacities of the Eca-109 and TE-1 cell lines (Figure 2C, D). Furthermore, UBE3A knockdown significantly inhibited esophageal cancer cell invasion (Figure 2E, F). Conversely, ectopic transfection with FLAG-UBE3A plasmids enhanced the protein and mRNA levels of UBE3A in Eca-109 and TE-1 cell lines (Figure 2G, H). This overexpressed UBE3A promoted proliferation and invasion by esophageal cancer cells *in vitro* (Figure 2I, J). Next, we used subcutaneously injected xenografts of esophageal cancer cells in nude mice to study the tumor-growth related effects of UBE3A *in vivo*. Stable UBE3A knockdown (shUBE3A) or UBE3A overexpression (Tsin-UBE3A) to restore UBE3A levels in Eca-109 cells were performed by using lentivirus-based constructs. Importantly, UBE3A knockdown impeded tumor growth* in vivo*, while UBE3A rescue enhanced tumor growth *in vivo* (Figure 2K-M). Thus, our results indicated that UBE3A was responsible for the cellular progression of esophageal cancer.

### UBE3A binds to ZNF185 and is negatively associated with the protein level of ZNF185

**Figure 3:**
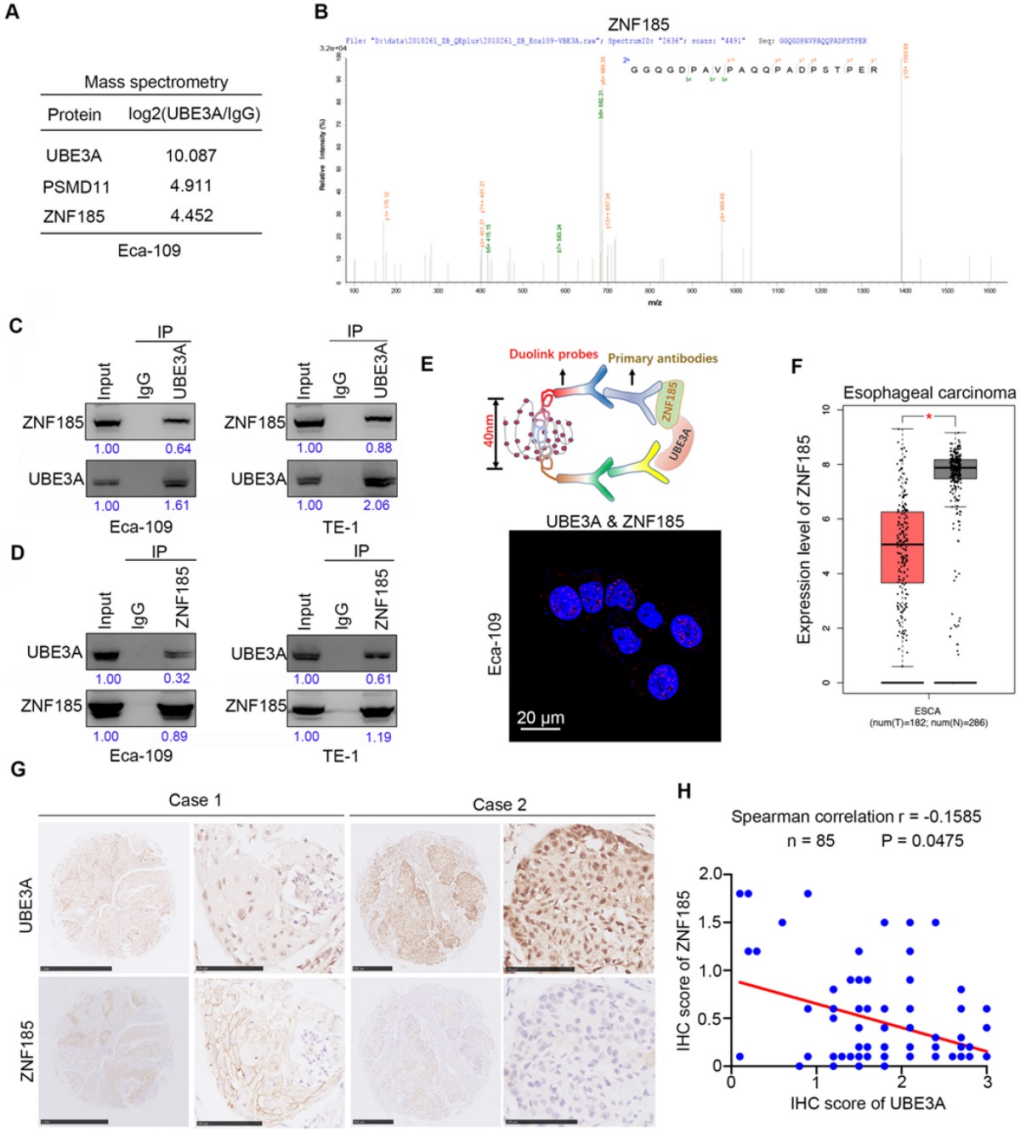
** UBE3A binds to ZNF185 and is negatively associated with the protein level of ZNF185. A and B,** the WCL of Eca-109 cells were subjected to mass spectrometry with IgG and UBE3A antibodies. **C and D,** Co-IP to detect the interaction between UBE3A and ZNF185. The WCL of Eca-109 and TE-1 cells was immunoprecipitated under nondenaturing condition with αUBE3A (C) or αZNF185 (D) and probed with αUBE3A or αZNF185 in Western blot. The protein levels of UBE3A or ZNF185 were quantified by using the Image J software. The quantified values of UBE3A or ZNF185 were normalized to the values of Input. **E,** the proximity ligation assay by using the indicated antibodies to verify the interaction between UBE3A and ZNF185 in Eca-109 cells. **F,** analysis the mRNA expression level of ZNF185 by using the GEPIA web tool (http://gepia.cancer-pku.cn/). **G and H,** the tissue microarray of esophageal cancer was stained with UBE3A and ZNF185 respectively. The typical IHC images stained with UBE3A and ZNF185 were shown in panel G. The correlation of these two proteins was shown in panel H, the P value was indicated in the figure.

To explore the molecular mechanism by which UBE3A promotes tumor progression, we performed mass spectrometry to identify proteins that interacted with UBE3A, using PSDM11 as a positive control 19 (Figure 3A). Our findings suggested that ZNF185 might interact with UBE3A (Figure 3A, B). Co-immunoprecipitation analyses demonstrated that UBE3A bound to ZNF185 in a reciprocal manner in both Eca-109 and TE-1 esophageal cancer cells (Figure 3C, D). We also confirmed the interaction between UBE3A and ZNF185 using a proximity ligation assay (Figure 3E). Subsequently, we examined the relationship between UBE3A and ZNF185. Notably, analysis with the Gene Expression Profiling Interactive Analysis (GEPIA) web tool 20 indicated that ZNF185 was downregulated in esophageal cancer tissues, compared with non-tumor esophageal tissues (Figure 3F). In consistent, ZNF185 mRNA levels were higher in esophageal cancer tissues than in non-normal tissues from resected esophageal cancer specimens in our hospital through RT-qPCR analysis (Supplementary Figure 1B). Surprisingly, UBE3A was not associated with ZNF185 at the mRNA level in an analysis of the TCGA dataset (Supplementary Figure 1C-F), although it was negatively associated with ZNF185 at the protein level in a TMA analysis using samples from patients with esophageal cancer (Figure 3G, H). Therefore, we concluded that UBE3A interacted with ZNF185 and was negatively associated with the protein level of ZNF185 in esophageal cancer.

### ZNF185 is degraded by UBE3A in esophageal cancer cells

**Figure 4:**
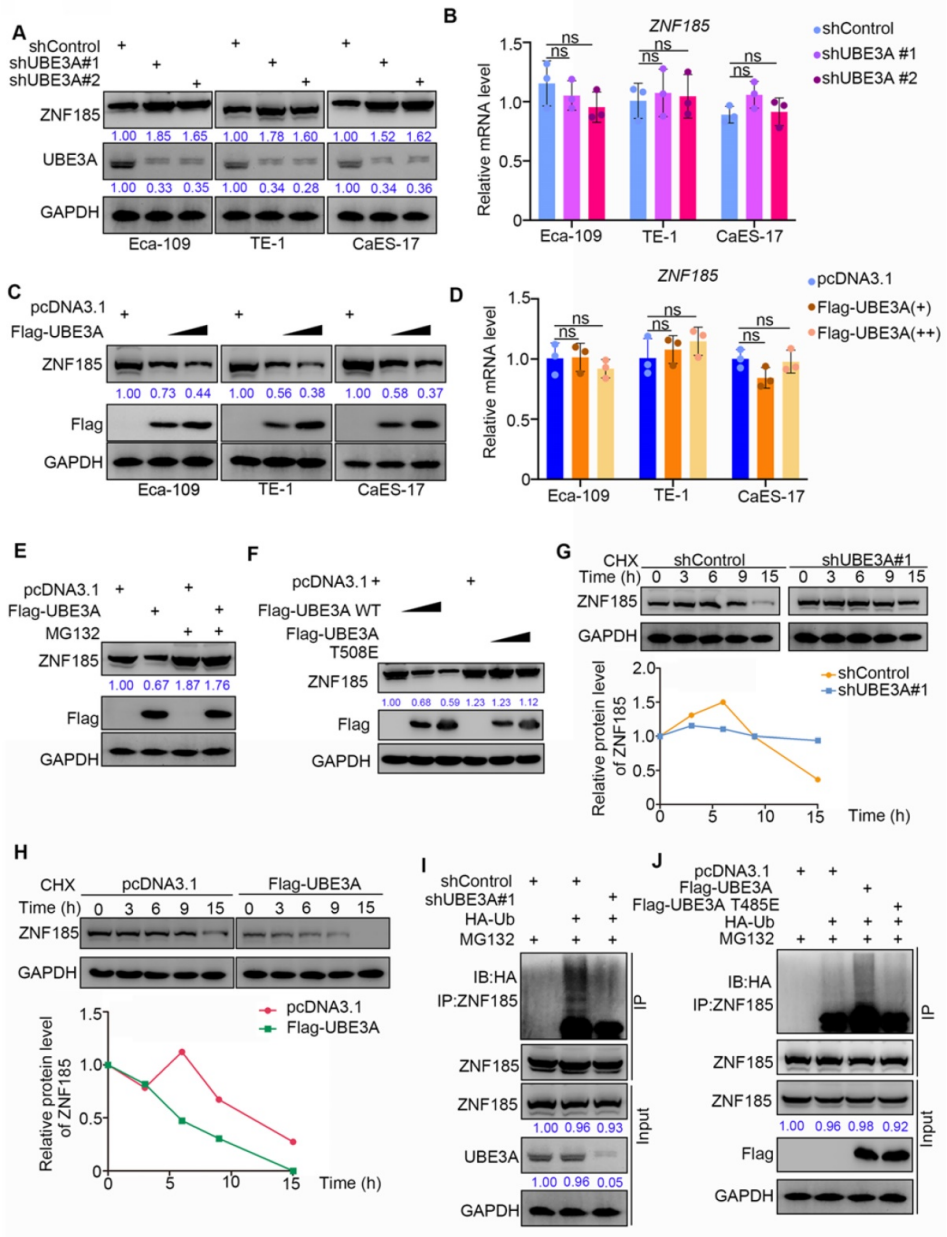
** ZNF185 is degraded by UBE3A in esophagus cancer cells. A and B, t**he esophageal cancer cell lines (Eca-109, TE-1 and CaES-17) were infected with indicated shRNAs. 72 h post infection, cells were harvested for Western blotting analysis (A) and RT-qPCR analysis (B). Data presented as Mean ± SD with three replicates. ns, not significant.** C and D,** the esophageal cancer cell lines (Eca-109, TE-1 and CaES-17) were transfected with indicated constructs. After 48 h, cells were harvested for Western blotting analysis (C) and RT-qPCR analysis (D). Data presented as Mean ± SD with three replicates. ns, not significant. **E,** Eca-109 cells were transfected with indicated constructs. After 48 h, the WCL of Eca-109 was subjected to Western blot analysis. Cells were treated with or without 20 µM of MG132 for 8 h before harvested. **F,** Eca-109 cells were transfected with indicated constructs. After 48 h, the WCL of Eca-109 was subjected to Western blot analysis. **G,** Eca-109 cells were infected with indicated shRNAs. After 72 h, cells were treated with Cycloheximide (CHX) and cells were collected for Western Blot analysis at different time points. **H,** Eca-109 cells were transfected with indicated plasmids. After 48 h, cells were treated with Cycloheximide (CHX) and cells were collected for Western Blot analysis at different time points. **I,** Eca-109 cells were infected with indicated plasmids. 72 h post-infection, cells were collected for Western Blot analysis after treated with MG132 for 8 h. **J,** Eca-109 cells were transfected with indicated plasmids. After 48 h, cells were collected for Western Blot analysis after treated with MG132 for 8 h.

Because UBE3A possesses an E3 ligase function, and the protein level of ZNF185 was negatively associated with UBE3A, we hypothesized that UBE3A might be involved in promoting the degradation of ZNF185 in esophageal cancer. To test this hypothesis, we performed UBE3A knockdown in three esophageal cancer cell lines (Figure 4A). UBE3A knockdown led to enhanced protein expression of ZNF185, but did not impact its mRNA expression, in all three esophageal cancer cell lines (Figure 4A, B). In contrast, UBE3A overexpression led to reduced protein expression of ZNF185, but did not impact is mRNA expression, in all three esophageal cancer cell lines (Figure 4C, D). Furthermore, we demonstrated that the reduced protein expression of ZNF185 in Eca-109 cells could be alleviated by treatment with a proteasome inhibitor (MG132) (Figure 4E). Similarly, the E3 ligase function negative mutant UBE3A T508E 21 could not decreased ZNF185, compared with wild-type UBE3A (Figure 4F). Moreover, we found that UBE3A knockdown extended the protein half-life of ZNF185 and decreased the level of ZNF185 ubiquitination in Eca-109 cells (Figure 4G, I). Conversely, UBE3A overexpression shortened the lifespan of ZNF185 protein and increased the level of ZNF185 ubiquitination in Eca-109 cells (Figure 4H, J). Together, these results suggested that ZNF185 is a degradation target of UBE3A in esophageal cancer cells.

### ZNF185 impedes the cellular progression of esophageal cancer

**Figure 5:**
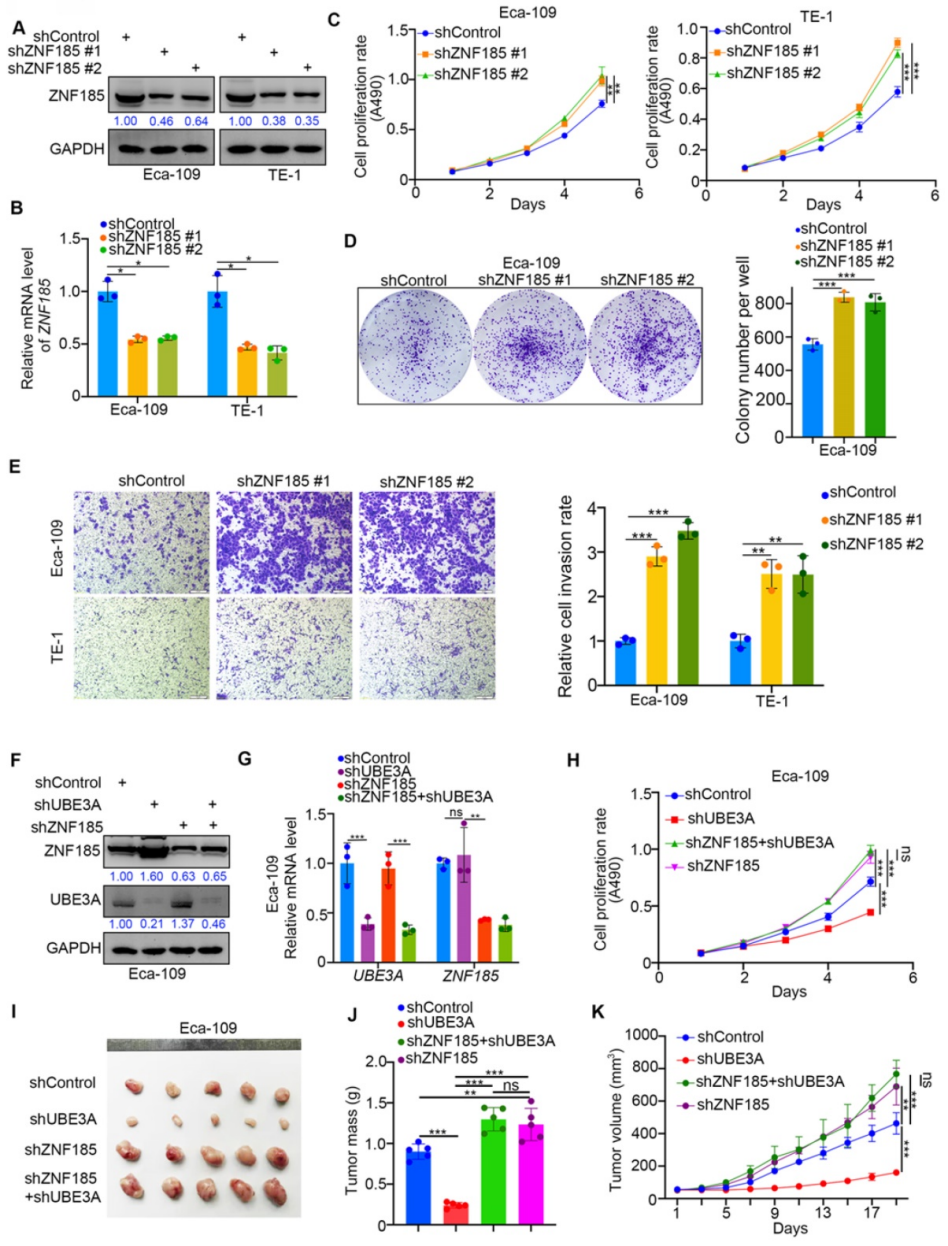
** ZNF185 impedes the cellular progression of esophageal cancer. A-E,** Eca-109 and TE-1 cells were infected with indicated shRNAs. 72 h post infection, cells were harvested for Western blotting analysis (A), RT-qPCR analysis (B), MTS assay (C), colony formation assay (D) and transwell assay (E). For the panel B-E, data presented as Mean ± SD with three replicates. *, P < 0.05; **, P < 0.01; ***, P < 0.001. **F-K,** Eca-109 cells were infected with indicated shRNAs. After 72h puromycine selection, cells were harvested for Western blot analysis (F), RT-qPCR analysis (G), MTS assay (H) and subcutaneously injected into nude mice for xenografts assay (I-K). The image of tumor was shown in panel I. The tumor mass was demonstrated in panel J. The tumor growth curve was indicated in panel K. For RT-qPCR and MTS assay, data presented as Mean ± SD with three replicates. Ns, not significant; **, P < 0.01; ***, P < 0.001. For mouse study, data presented as Mean ± SD with five replicates. *, P < 0.05; **, P < 0.01; ***, P < 0.001.

Because UBE3A promoted the progression of esophageal cancer cells, we investigated whether ZNF185 influenced the UBE3A-induced tumor growth in esophageal cancer cells. We first performed ZNF185 knockdown in both Eca-109 and TE-1 cells (Figure 5A, B). MTS and colony formation assays showed that ZNF185 knockdown significantly enhanced the proliferation of esophageal cancer cells (Figure 5C, D). While, ZNF185 overexpression decreased the cell proliferation ability in both Eca-109 and TE-1 cells (Supplementary Figure 1G, H). Additionally, ZNF185 knockdown greatly enhanced the invasiveness of esophageal cancer cells (Figure 5E). Subsequently, we compared the effects of UBE3A and ZNF185 knockdown, alone and in combination, in Eca-109 cells (Figure 5F, G). We showed that the inhibition of tumor growth caused by UBE3A knockdown could be attenuated by concurrent knockdown of ZNF185, both *in vitro* and *in vivo* (Figure 5H-K). These data indicated that ZNF185 is responsible for modulating the UBE3A function in esophageal cancer cells.

### UBE3A activates the NOTCH pathway via ZNF185 in esophageal cancer cells

**Figure 6:**
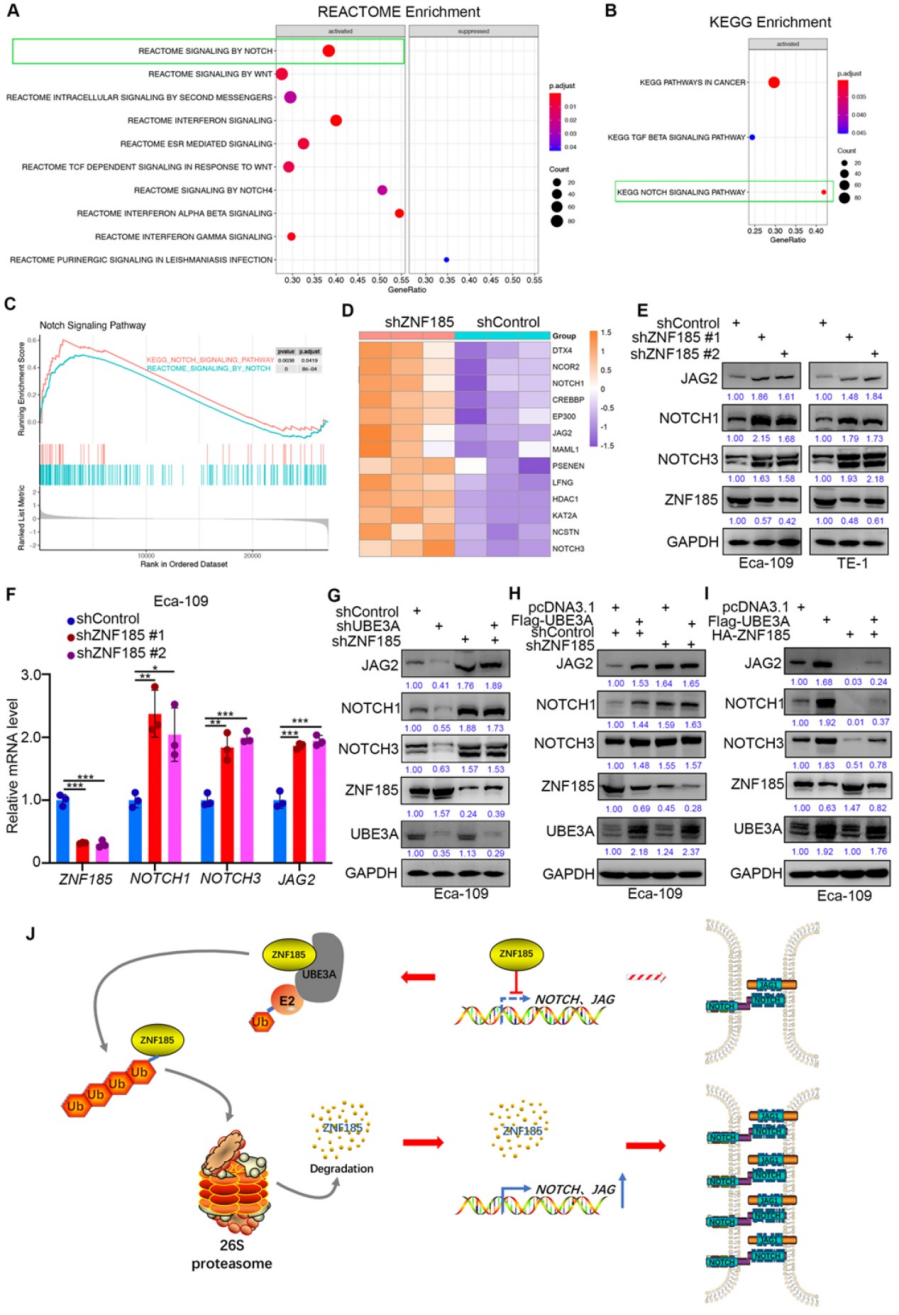
** UBE3A activates NOTCH pathway via ZNF185 in esophagus cancer cells. A and B,** Eca-109 cells were transfected with indicated constructs for 48h. Cells were subjected to RNA-seq analysis and subsequent Reactome (A) and KEGG pathway enrichment (B). **C and D,** Notch signaling pathway was activated after knockdown of ZNF185 (C). The heatmap indicated genes involved in Notch signaling pathway changed after knockdown of ZNF185 (D). **E and F,** Eca-109 and TE-1 cells were infected with indicated shRNAs. 72 h post infection, cells were harvested for Western blotting analysis (E) and RT-qPCR analysis (F). **G,** Eca-109 cells were infected with indicated shRNAs. 72 h post infection, cells were harvested for Western blotting analysis. **H,** Eca-109 cells were infected with indicated shRNAs. After 48 h, cells were transfected with indicated plasmids. 24 h post transfection, cells were harvested for Western blotting analysis. **I**, Eca-109 cells were transfected with indicated plasmids for 24 h. Cells were harvested for Western blotting analysis. **J,** The hypothesis model depicts that ZNF185 inhibits the NOTCH pathway in esophageal cancer, then the up-regulated UBE3A promotes ZNF185 degradation to activate the NOTCH signaling pathway in esophageal cancer cells.

Finally, we investigated the mechanism by which ZNF185 contributes to the suppression of esophageal cancer, using a transcriptome sequencing approach in a ZNF185 knockdown line of Eca-109 cells. REACTOME and KEGG analyses of the RNA-seq data indicated that ZNF185 contributed to inhibition of the NOTCH signaling pathway (Figure 6A, B). Furthermore, the REACTOME and KEGG analyses of the TCGA dataset showed that ZNF186 regulated the NOTCH pathway in esophageal cancer (Figure 6C). Our RNA-seq data revealed that ZNF185 knockdown led to the upregulation of many NOTCH pathway-related proteins (Figure 6D). Moreover, the upregulation of NOTCH1, NOTCH3 and JAG2 were confirmed in both Eca-109 and TE-1 cells (Figure 6E, F). Furthermore, we demonstrated that UBE3A knockdown reduced the expression levels of NOTCH1, NOTCH3, and JAG2; these changes could be attenuated by concurrent ZNF185 knockdown in Eca-109 cells (Figure 6G). Conversely, UBE3A overexpression enhanced the expression levels of NOTCH1, NOTCH3, and JAG2; ZNF185 knockdown diminished these effects (Figure 6H). Moreover, we also demonstrated that ZNF185 overexpression decreased the expression levels of NOTCH1, NOTCH3, and JAG2 in Eca-109 cells (Figure 6I). Meanwhile, due to UBE3A degrading ZNF185, co-overexpression of ZNF185 and UBE3A attenuated the down-regulation effect of NOTCH1, NOTCH3, and JAG2 induced by ZNF185 overexpression alone (Figure 6I). Taken together, our data suggested that UBE3A activates the NOTCH pathway via ZNF185 in esophageal cancer cells.

## Discussion

ZNF185 is a member of the zinc finger protein family that participates in male reproduction by regulating the secretion of testosterone 22. Additionally, ZNF185 contains the LIM domain, which is involved in multiple fundamental biological processes 23. ZNF185 expression is reportedly an independent predictor of both prognosis and liver metastasis in patients with colon cancer 23. Bioinformatics analyses of TCGA and GTX datasets has shown that ZNF185 expression is reduced in various types of epithelial cancer. Furthermore, ZNF185 overexpression inactivates the AKT/GSK3β signaling pathway, thus inhibiting cell proliferation and invasion in lung adenocarcinoma 24. Importantly, the role of ZNF185 in pancreatic cancer is dependent on its subcellular localization. Expression of ZNF185 at the plasma membrane is associated with an unfavorable prognosis in patients with pancreatic cancer 25. Moreover, ZNF185 contributes to chemotherapy resistance, in combination with elevated SMAD4 expression, in pancreatic cancer 26. Here, our results suggested that ZNF185 knockdown led to enhanced cell proliferation among esophageal cancer cells by activation of the NOTCH pathway. Thus far, the role of ZNF185 in cancers remains controversial. To the best of our knowledge, the regulation of ZNF185 has not been extensively investigated. p53 and p63 have been shown to enhance ZnF185 expression at the transcriptional level, thereby regulating the DNA damage response and development of squamous cell carcinoma 27, 28. In this study, we explored the mechanism underlying post-transcriptional regulation of ZNF185; we found that ZNF185 underwent ubiquitin-dependent proteasome degradation in the presence of UBE3A in esophageal cancer cells.

UBE3A has an important viral oncogenesis function in human papillomavirus-induced tumors, which is mediated by degradation of the tumor suppressor protein p53 15. Studies of UBE3A in non-viral oncogenesis cancers have provided useful insights regarding its role in tumors. The biological role of UBE3A varies among cancer types. In its E3 ligase capacity, UBE3A reportedly interacts with the tumor suppressor promyelocytic leukemia protein (PML) in leukemia, Burkitt's lymphoma, and prostate cancer 29-31. The tumor suppressor protein p27^Kip1^ is also a target for UBE3A-induced ubiquitin-dependent degradation in prostate cancer 31. In contrast, UBE3A binds with ER-α to promote ER-α proteasome degradation; reduced expression of UBE3A has been associated with poor prognosis in human invasive breast cancers 32. Moreover, UBE3A reportedly causes reduced expression of the oncogenic proteins AIB1 (amplified in breast cancer 1) and Enolase1 (ENO1) in breast cancer cells 33. In this study, we demonstrated that UBE3A was upregulated in esophageal cancer. This upregulated UBE3A bound to the tumor suppressor ZNF185, initiating the ubiquitin-dependent degradation of ZNF185. Thus, UBE3A might act as an oncogenic protein in esophageal cancer cells. Furthermore, the non-E3 ligase function of UBE3A has been reported previously. Nawaz et al. reported that UBE3A is a transcriptional co-activator of AR, PR, and ER-α; it is involved in the tumorigenesis of prostate and breast cancer 34. Therefore, we presume that the function of UBE3A varies according to tumor type; its functions in specific tumors should be further explored.

## Conclusion

Collectively, our results suggest that UBE3A is aberrantly upregulated in specimens from patients with esophageal cancer. This upregulated UBE3A participates in the cellular progression of esophageal cancer. Furthermore, we found that UBE3A promotes ZNF185 degradation to activate the NOTCH signaling pathway in esophageal cancer cells (Figure 6J). Taken together, our data indicate that the UBE3A/ZNF185/NOTCH axis is important in the progression of esophageal cancer.

## Supplementary Material

Supplementary figure, materials and methods, tables.

## Abbreviations

UBE3A: Ubiquitin-protein ligase E3A
ZNF185: Zinc finger protein 185
NOTCH: Neurogenic locus notch homolog protein
PLA: Proximity ligation assay
IP: immunoprecipitated
GEPIA: Gene Expression Profiling Interactive Analysis
