# Supplementary material for: UBE3A activates the NOTCH pathway and promotes esophageal cancer progression by degradation of ZNF185
Source: Int J Biol Sci. 2021 Jul 13;17(12):3024–35. doi: 10.7150/ijbs.61117 (PMC8375240; doi:10.7150/ijbs.61117)
Supplement: Supplementary file 1 — Supplementary figure, materials and methods, tables. [file ijbsv17p3024s1.pdf]

UBE3A activates the NOTCH pathway and promotes esophageal cancer progression by degradation of ZNF185

Zhikun Zheng, Bin Zhang, Haixin Yu, Shoukang Li, Naicheng Song, Xin Jin, Jinsong Li

Supplementary Figure 1

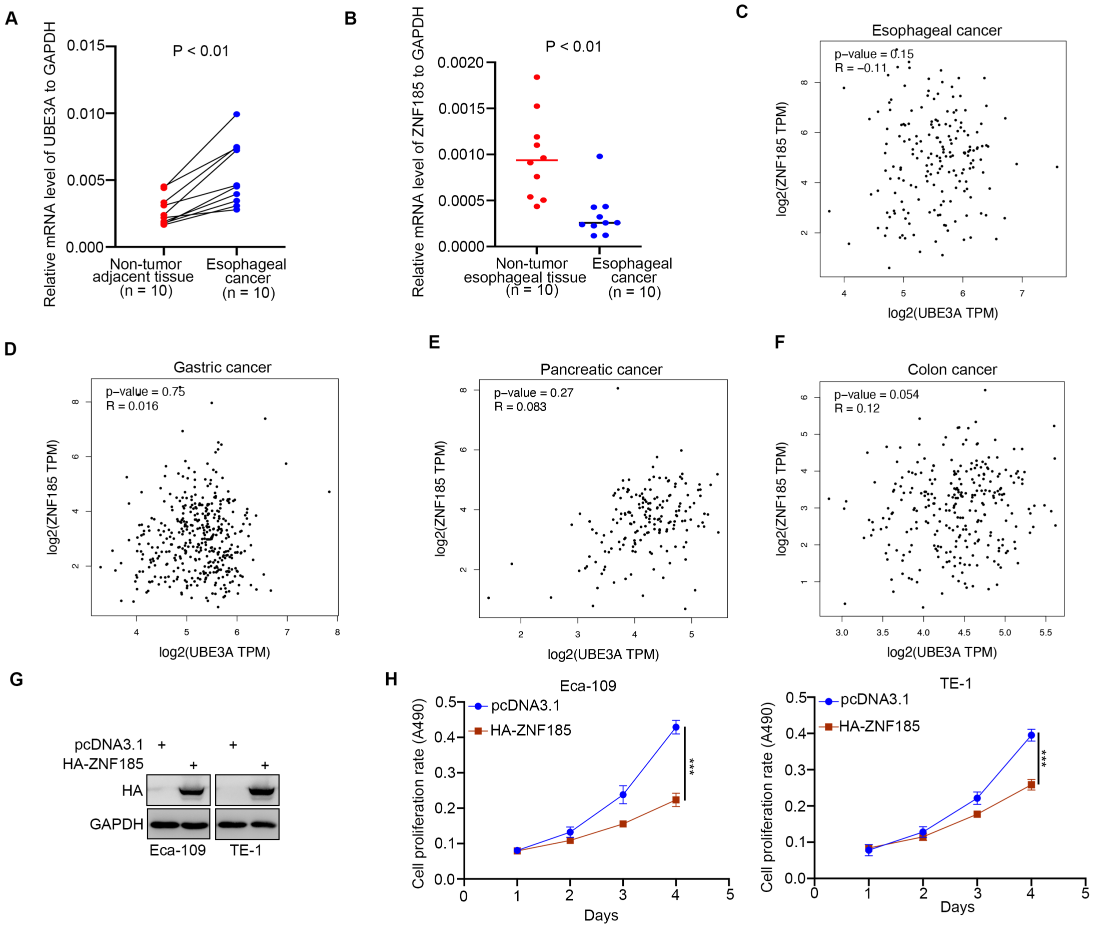

Supplementary Figure 1. A and B, the mRNA level of UBE3A (A) and ZNF185 (B) from esophageal cancer tissues (n = 10) and adjacent normal esophagus tissues (n = 10) was detected by RT-qPCR analysis, P < 0.01 **.C-F,** Analyzing the spearman correlation between the mRNA level of UBE3A and ZNF185 in esophageal cancer (C), gastric cancer (D), pancreatic cancer (E) and colon cancer (F) by using the GEPIA web tool. **G and H**, Eca-109 and TE-1 cells were transfected indicated constructs. After 24 h, cells

were harvested for Western blotting analysis (G), and MTS assay (H). Data presented as Mean ± SD with three replicates. ***, $P < 0.001$.

Supplementary Material and Methods

Cell proliferation assay

Cell proliferation was determined using an MTS assay. Esophageal cancer cells (1×10^4 cells/well) were seeded in 96-well plates and incubated for 24h. Then, the medium was replaced with fresh medium containing MTS reagent (Cat. No. ab197010, Abcam), and the absorbance of each well at 490 nm was determined the cell growth ability.

For colony formation assay, esophageal cancer cells (500 cells/well) were cultivated into 6-well plates and incubated in RPMI-1640 medium with 10% FBS at 37°C for 2 weeks. Then the cells were fixed in methanol for 30 min and stained with 1% Crystal Violet Staining Solution for 30 mins and then washed with PBS 3 times. Finally, the number of colonies was calculated.

Quantitative real-time PCR (qRT-PCR)

Total RNA from pancreatic cancer cells was isolated using TRIzol reagent (Invitrogen) according to the manufacturer's instructions. And cDNA was synthesized using the cDNA Reverse Transcription kit (PrimeScript™ RT reagent Kit). The qRT-PCR analysis was conducted using a PCR kit (TB Green™ Fast qPCR Mix). Relative mRNA levels of target genes were calculated using the $2^{-\Delta\Delta Cq}$ method after

normalization to *GAPDH* mRNA levels. The sequences of primers were provided in Table S1.

Cell invasion assay

The in vitro cell invasion assay was applied by using a Bio-Coat Matrigel invasion chamber (BD Biosciences). Each transwell chamber was coated with 50 μL matrigel (1: 8, CORNING). Cells with 100 μL serum-free RPMI-1640 medium were incubated in the upper chamber for 24 h (lower chamber containing complete medium). Then cells were fixed in methanol for 15 min and then stained with 1% crystal violet for 20 min. Cell images were taken in three fields under the microscope, and the number of cells penetrating the membrane was counted.

Table S1: Sequences of RT-qPCR primers

**Species**	**Gene**	**Forward (5'-3')**	**Reverse (5'-3')**
Human	*GAPDH*	CCAGAACATCATCCCTGCCT	CCTGCTTCACCACCTTCTTG
Human	*UBE3A*	CCCTGATGATGTGTCTGTGG	GGCAAAGCCATTTCCAGATA
Human	*ZNF185*	ACAATATCAGGCGCAGCTCT	GTGTCTCCTGGGTAGGACCA
Human	*NOTCH1*	ACTGTGAGGACCTGGTGGAC	TTGTAGGTGTTGGGGAGGTC
Human	*NOTCH3*	TGTGGACGAGTGCTCTATCG	AATGTCCACCTCGCAATAGG
Human	*JAG2*	AGGTGGAGACGGTTGTTACG	TTGCACTGGTAGAGCACGTC

Table S2: Sequences of gene-specific shRNAs

shUBE3A-1	5′- CCGGCCTACATCTCATACTTGCTTTCTCGAGAAAGCAAGTATGAGATGTAGGTTTTT-3′
shUBE3A-2	5′- CCGGCCTGATGATGTGTCTGTGGATCTCGAGATCCACAGACACATCATCAGGTTTTT-3′
shZNF185-1	5′- CCGGCCCTGCTGATAGGAAGAGCAACTCGAGTTGCTCTTCCTATCAGCAGGGTTTTTG-3′
shZNF185-2	5′- CCGGCCAAAGATTACCCTAGAACATCTCGAGATGTTCTAGGGTAATCTTTGGTTTTTG-3′
